# Knowledge, Attitudes, Practice, and Hesitancy of Patients and HCWs Towards COVID-19 Vaccination and Factors Associated with Vaccination in the Republic of Srpska, Bosnia and Herzegovina

**DOI:** 10.3390/epidemiologia7010012

**Published:** 2026-01-12

**Authors:** Biljana Mijović, Tihomir Dugandžija, Dragana Sokolović, Dragana Drakul, Jovan Kulić, Kristina Drašković Mališ, Anđela Bojanić, Nasta Manojlović, Milena Dubravac Tanasković, Marija Milić, Radmila Balaban-Đurević, Dajana Nogo-Živanović, Slađana Mihajlović, Bojan Joksimović

**Affiliations:** 1 Faculty of Medicine Foča, University of East Sarajevo, 73300 Foča, Bosnia and Herzegovina; biljana.mijovic@ues.rs.ba (B.M.); dragana.sokolovic@ues.rs.ba (D.S.); dragister@gmail.com (D.D.); kulicjovan@yahoo.com (J.K.); kristina.dr995@gmail.com (K.D.M.); radmilabalaban@yahoo.com (R.B.-Đ.); dajana.nogozivanovic@ues.rs.ba (D.N.-Ž.); bojannjoksimovic@gmail.com (B.J.); 2 Faculty of Medicine, University Novi Sad, 21000 Novi Sad, Serbia; tihomir.dugandzija@mf.uns.ac.rs; 3 Centre for Biomedical Research, Faculty of Medicine, University of Banja Luka, 78000 Banja Luka, Bosnia and Herzegovina; andjela.bojanic@med.unibl.org; 4 PHI Hospital “Sveti Vračevi” Bijeljina, 76300 Bijeljina, Bosnia and Herzegovina; nasta.manojlovic@bolnicabijeljina.com; 5 Faculty of Medicine, University of Priština Temporarily Settled in Kosovska Mitrovica, 38220 Kosovska Mitrovica, Serbia; marijamilic85@gmail.com; 6 Clinical Hospital Centre “Dragiša Mišović Dedinje”, 11000 Belgrade, Serbia; drsladjanamihajlovic@gmail.com; 7 Faculty of Medicine, University of Belgrade, 11000 Belgrade, Serbia

**Keywords:** KAP study, COVID-19 vaccination, patients, healthcare workers, hesitancy

## Abstract

Background/Objectives: The COVID-19 pandemic caused over seven million deaths globally as of July 2024. In an attempt to bring the pandemic under control, immunization was implemented as the main preventive strategy. This study aimed to investigate the knowledge, attitudes, and practices (KAP) of hospitalized patients and healthcare workers (HCWs) regarding COVID-19 vaccination, as well as the factors contributing to COVID-19 vaccination rates. Methods: This cross-sectional, survey-based KAP study was conducted between November 2024 and February 2025 in five hospitals across five cities of the Republic of Srpska, Bosnia and Herzegovina. Results: There were 571 respondents, 68% of whom were female, with an average age of 39.17 ± 14.74 years; one-third held a university degree. The study sample consisted of patients and healthcare workers (HCWs) (59% vs. 41%). During the pandemic period, 46.6% of respondents were diagnosed with COVID-19, with a higher prevalence among healthcare workers compared to patients (54.2% vs. 41.2%). Among the 55.2% of respondents who were vaccinated, HCWs were more often vaccinated than patients (70.9% vs. 44.2%) and more likely to know that vaccines protect against severe forms of disease and death (80.8% vs. 68.5%). Patients more often believed that vaccination against COVID-19 may lead to sterility in young patients (11.3% vs. 6%) and were more often afraid of vaccination compared to the occurrence of COVID-19 (35.6% vs. 24.8%). Regression analyses showed that independent predictors of COVID-19 vaccination were older age (*p* < 0.001), higher education level (*p* = 0.039), knowledge of vaccine production technology, and the belief that vaccinated individuals have milder symptoms of the disease *(p* = 0.002). Conversely, the belief that the COVID-19 situation was overblown was negatively associated with vaccination (*p* = 0.004). Conclusions: HCWs had better knowledge, more positive attitudes, and better vaccination practices against COVID-19 in comparison to patients. However, there are still certain dilemmas and hesitations among HCWs toward COVID-19 vaccination.

## 1. Introduction

COVID-19, caused by Severe Acute Respiratory Syndrome Coronavirus 2 (SARS-CoV-2), quickly became a public health threat worldwide after its emergence in China in 2019. The World Health Organization (WHO) declared a public health emergency of international concern by the end of January 2020 and urged all governments to work together to prevent its rapid spread [1]. As of 14 July 2024, SARS-CoV-2 has resulted in 777,686,716 cases and 7,054,093 deaths worldwide [2]. Global immunization represents one of the most effective preventive strategies in the management of COVID-19. Vaccination has been shown not only to reduce the incidence of symptomatic infections but also to lower hospitalization and mortality rates associated with SARS-CoV-2 infection. Therefore, widespread vaccination remains a critical tool in limiting severe clinical outcomes and alleviating the burden on healthcare systems. Furthermore, achieving high levels of vaccine coverage contributes to community protection, curtails viral transmission, and reduces the likelihood of additional mutations [3]. The emergence of novel SARS-CoV-2 variants has posed significant challenges to global public health. Mutations, particularly within the spike protein, may compromise the effectiveness of existing vaccines by enabling partial immune escape. In regions where vaccination coverage remains low, such variants are afforded greater opportunity to disseminate, thereby amplifying transmission dynamics and contributing to increased case prevalence [4]. As of 31 December 2023, a total of 13.64 billion doses of COVID-19 vaccines had been administered worldwide. A complete primary series of the COVID-19 vaccine had been received by 67% of the global population, while 32% had received at least one booster dose [5].

**Figure 1:**
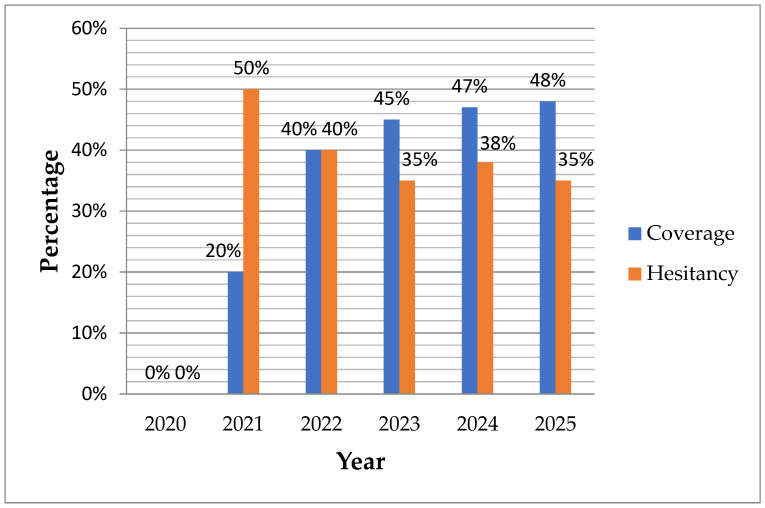
Annual COVID-19 vaccination coverage and hesitancy trends, 2020–2025.

Over the course of one year, from 12 February 2021 to 12 February 2022, a total of 719,224 doses of COVID-19 vaccines were administered to residents of the Republic of Srpska, equating to 62,655 doses per 100,000 inhabitants. According to the PHI RS report, the most frequently administered COVID-19 vaccines were Sinopharm (38.1%), Pfizer/BioNTech (27.1%), Sputnik V (26.6%), AstraZeneca (5.2%), Sinovac (2.9%), and Moderna (0.1%). As of 12 February 2022, vaccination coverage among citizens aged 16 and older was approximately 40%, with the highest coverage among older adults (62% of residents aged 65–79 had completed the primary two-dose course), and significantly lower coverage among younger individuals and children (13.8% for those aged 20–29, 3.9% for those aged 15–19, and 0.2% for those aged 5–14) (Official Report on COVID-19 Vaccination, Institute of Public Health of the Republic of Srpska, unpublished). In 2024–2025, during the study period, global COVID-19 vaccination coverage and hesitancy demonstrated considerable regional disparities, with around 21% of HCWs reporting strong or moderate COVID-19 vaccine hesitancy [6,7]. Locally, in the Republic of Srpska, coverage was ~48%, with hesitancy at 35–45% due to ongoing misconceptions [8]. Figure 1 illustrates the annual trends in COVID-19 vaccination coverage and hesitancy in the Republic of Srpska from 2020 to 2025, demonstrating post-2022 stabilization and alignment with the study’s 55.2% sample vaccination rate (Figure 1).

With the expansion of COVID-19 vaccination, misinformation and fears also spread, leading to hesitancy and opposition to these vaccines. Research indicates that people refused the COVID-19 vaccine due to doubts about its effectiveness and safety, as well as a lack of trust in government or healthcare systems [5,9,10,11,12]. According to the findings of a systematic review of the literature conducted by Alhumaid et al. [13], there was significant hesitancy regarding the COVID-19 vaccine. The perceived effectiveness and safety of the vaccine were lower than indicated by clinical data. Distrust in the vaccines, their manufacturers, various institutions, and governments, along with personal beliefs and feelings, age, gender, education, and socioeconomic status, were identified as factors influencing attitudes towards COVID-19 vaccination [14].

A large number of studies have investigated the knowledge, attitudes, and practices regarding COVID-19 vaccination among healthcare workers (HCWs), medical students, and various patient categories [14,15,16,17,18,19,20]. However, only a small number of studies have examined the differences in COVID-19 vaccination hesitancy between healthcare and non-medical individuals. In a study conducted by Aloweidi et al. [21] at the University Hospital in Jordan in early 2021 among field service employees, 35% of surveyed HCWs and only 26.2% of surveyed non-medical individuals stated they would accept COVID-19 vaccination. The study does not provide evidence of differences in factors contributing to COVID-19 vaccination hesitancy between HCWs and patients [21]. However, in the review paper by Joshi et al. [22], gender, age, education level, and occupation were some of the socio-demographic variables associated with COVID-19 vaccine acceptance. Factors influencing vaccine rejection included distrust in the authorities, perception of the risk of COVID-19 infection, vaccine efficacy, current or previous flu vaccination, and vaccine safety [22].

This study aimed to investigate the knowledge, attitudes, and practices (KAP) of hospitalized patients and HCWs regarding COVID-19 vaccination, as well as the factors contributing to COVID-19 vaccination rates.

## 2. Materials and Methods

### 2.1. Settings

A cross-sectional study was conducted among 571 HCWs and patients in five hospitals of secondary level across five cities in the eastern part of the Republic of Srpska (RS), Bosnia and Herzegovina (Doboj, Zvornik, Istočno Sarajevo, Foča, and Trebinje), from 1 November 2024 to 31 January 2025.

### 2.2. Participants

The sample included all HCWs and hospitalized patients present in the hospitals on the days of the research who providedinformed consent. Firstly, on the day of the study, we identified how many patients and HCWs were present in the each hospital. Secondly, we selected patients and HCWs who met the inclusion criteria, and lastly, we included all patients and HCWs that met the inclusion criteria and agreed to participate in the study. The study sample was selected as shown in Table 1.

Participation required written informed consent from all subjects, obtained prior to questionnaire administration, in accordance with the Declaration of Helsinki (Ethics approval: protocol 01-2-30, 19 March 2024). Age restrictions: Participants were adults aged ≥ 19 years (as per sample demographics: mean age 39.18 ± 14.73; youngest group 19–45 years, Table 2). No minors (<18 years) were included; therefore, no special procedures for parental/guardian consent or assent were needed.

The inclusion criteria for the patients were hospitalization on the day of the study, while the exclusion criteria included the following: outpatient examinations (ambulatory visits), surgery on the day of the study, inability to communicate due to his/her health condition (intubated, acute phase of stroke, etc.), and undergoing some diagnostic procedure outside the hospitalization departmentat the time of the study. Inclusion criteria for HCWs were as follows: working in a hospital on the day of the study. Exclusion criteria included the following: working in polyclinic services on the day of the study, performing operative procedures, or being engaged in emergency transport on that day.

The total number of patients (n = 593) and HCWs (n = 735) were taken into consideration as the target referent populations. The minimal sample size was determined to be 233 for patients, and 234 for HCWs, based on the reference population, assuming a 5% margin of error and a 95% confidence level. The actual sample size of 571 exceeded the minimum required sample of 296 (calculated using Raosoft, SurveyWin version 4.5, with a population of 1328, 5% margin of error, 95% confidence level, and 50% response distribution), ensuring representativeness. A post hoc power analysis for the significant predictor “older age” (*p* < 0.001) yielded a power greater than 0.90, confirming sufficient statistical power [23].

However, our patient sample was expanded, as we included all participants who met the inclusion criteria and agreed to participate in the study, while the number of HCWs remained the same as calculated.

### 2.3. Ethics Statements

The study was conducted in accordance with the Declaration of Helsinki and approved by the Institutional Ethics Committee of the Faculty of Medicine Foča, University of East Sarajevo (protocol code 01-2-30 and date of approval: 19 March 2024). Written informed consent was obtained from all participants involved in the study.

### 2.4. Face Validity, Internal Consistency and Overall Intelligibility of a Research Instrument

The research instrument was a survey questionnaire consisting of 59 questions, divided into the following sections: demographic data; experience with COVID-19; knowledge of COVID-19 vaccines; attitudes towards COVID-19 and the COVID-19 vaccines; COVID-19 vaccination practices; and hesitancy towards receiving the COVID-19 vaccine. The questionnaire was specially created regarding patients’ and HCWs’ knowledge, attitudes, and practices about COVID-19 vaccination, based on the questionnaires used in the cited studies.

The authors worked with two multilingual specialists to translate questions using the forward–backward method. Initially, the authors selected questions from the cited studies in English language, and a Serbian-speaking expert interpreted them into Serbian. The second expert then performed a reverse translation into English. Finally, the authors and specialists compared the two versions and introduced the Serbian version of the questionnaire.

At this point, the questionnaire was first distributed to 25 patients and 25 HCWs using convenience sampling to ensure face validity. Subsequently, the participants were asked to rate each item on a 5-point Likert scale, from 1 (not important at all) to 5 (very important), in relation to issues, ambiguity, relativity, proper terminology and grammar, and understandability [24]. Following the collection and analysis of all the questionnaires, the impact score for each item was calculated using the formula below, with scores greater than 1.5 considered acceptable (Frequency (%) × Importance = the impact score) [24]. Every participant, from both examined populations reported that each item on the questionnaire was straightforward, understandable, and relevant to the study objectives. Furthermore, all items had effect scores exceeding 1.5.

The internal consistency of the questionnaire (Cronbach’s alpha method) was used to evaluate its reliability. Cronbach’s alpha coefficients greater than 0.7 represented acceptable reliability. As a result, for each of the questionnaire’s sections, Cronbach’s alpha ranged from 0.86 to 0.94 in a patient group, and from 0.85 to 0.96 in HCWs group of respondents, with attitudes towards COVID-19 and COVID-19 vaccination practices having the highest reliability in both groups.

Additionally, a small independent sample from both groups (25 respondents per group) was asked to rate each question on a 7-point scale, from 1 (not meaningful at all) to 7 (extremely meaningful). This allowed the intelligibility of the questions to be assessed. Ten adjunct questions (AQs), which reported grammatical and semantic problems in addition to the standard questions (SQ), were added to the initial questionnaire, in order to achieve this goal. All SQs had mean scores greater than 6, and all AQs had mean scores less than 2. As a result, respondents from both groups considered the questionnaire’s content to be clear.

### 2.5. Statical Analysis

The distribution of data is evaluated using the Shapiro–Wilk test. Measures of central tendency and measures of variability (arithmetic mean, M) with standard deviation (±SD), and relative numbers were used for descriptive statistics. Mann–Whitney test and the Chi-square test were used to examine differences in frequency between groups. An association between various variables and COVID-19 vaccination status were assessed using odds ratio (OR) with 95% confidence intervals (95% CI), and *p* value was derived from binary multivariate logistic regression analysis. The level of statistical significance was set at *p* < 0.05. All statistical analyses were conducted using IBM SPSS Statistics Software version 24.0 for Windows (IBM Co., Armonk, NY, USA).

## 3. Results

### 3.1. Description of the Study Sample

Out of the total number of respondents, 337 (59%) were patients, while 234 (41%) were HCWs. Of the total number, 315 (55.2%) participants were vaccinated against COVID-19. The majority of respondents were women (68.3%), who were significantly more frequently represented among HCWs compared with men. The average age of the participants was 39.18 ± 14.73. HCWs and vaccinated participants were significantly older than unvaccinated ones, and they were significantly more likely to have completed university compared to patients and unvaccinated (Table 2).

### 3.2. Vaccination Status Among HCWs

**Figure 2:**
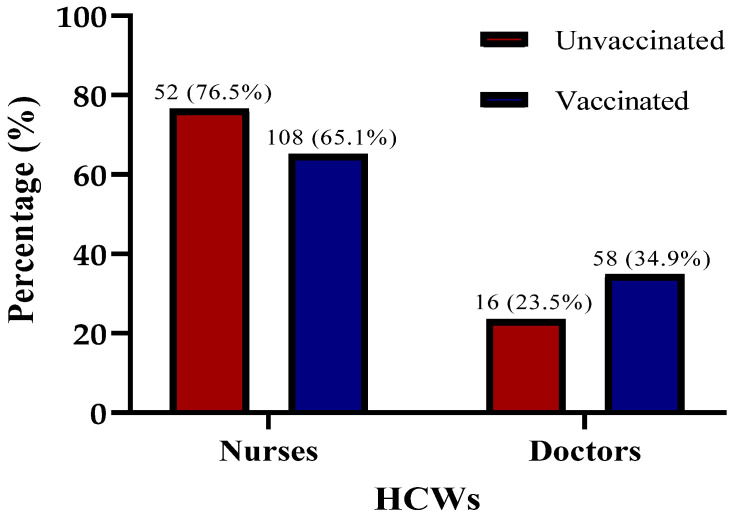
Distribution of HCWs (n = 234) according to their profession and their COVID-19 vaccination status. HCWs—healthcare workers. Statistical significance was measured by χ^2^—chi square test (*p* = 0.137).

Out of total 234 HCWs, 160 (68.4%) were nurses and 74 (31.6%) were medical doctors. There was no difference in COVID-19 vaccination status between HCWs when comparing these two professional groups (Figure 2).

### 3.3. Experience with COVID-19

HCWs (54.3% vs. 41.5%) and vaccinated (54.9% vs. 36.7%) respondents more often had confirmed case of COVID-19 in comparison to patients and unvaccinated individuals. PCR test for confirmation of COVID-19 was more often used in vaccinated respondents (59.5% vs. 46.8%). The symptoms of COVID-19 in the period before COVID-19 vaccination were more often present in HCW (59.6% vs. 44.4%) and vaccinated respondents (57.7% vs. 41%). Only 12.7% of respondents with confirmed COVID-19 were hospitalized at the time of diagnosis, with a mean hospitalization duration of 2.54 ± 3.18 days (Table 3).

### 3.4. Knowledge and Attitudes About COVID-19 Vaccination

Respondents’ level of knowledge of the availability of various COVID-19 vaccines was high for the Russian vaccine (Sputnik V) (93%), Chinese vaccine (Sinopharm and Sinovac) (89%), and Pfizer vaccine (BNT162b2) (88.3%), while the knowledge of availability of AstraZeneka vaccine (AZD122) (68%) and Moderna vaccine (mRNA-1273) was lower (57.1%). Furthermore, 55.7% of respondents knew that all of the aforementioned COVID-19 vaccines were available. Vaccinated respondents demonstrated better knowledge than patients and unvaccinated individuals regarding the following: Chinese vaccines were produced in the classic way as inactivated vaccines (65.7% vs. 52%); Pfizer vaccine uses RNA technology to induce immune response (47% vs. 36.7%); Pfizer and Moderna vaccines lead to the best immune response against COVID-19 (28.6% vs. 19.1%; *p* < 0.001). HCWs demonstrated better knowledge than patients regarding (51.3% vs. 36.2%) the technology behind Pfizer vaccines. However, 73.6% of respondents knew that COVID-19 vaccines protect against severe forms of disease and death, with HCWs (80.8% vs. 68.5%) and vaccinated respondents (78.4% vs. 67.6%) showing better knowledge than patients and unvaccinated respondents. When it comes to attitudes of respondents about COVID-19 vaccination, 67.8% states that the story about COVID-19 is overblown, significantly more often patients than HCWs (72.1% vs. 61.5%) and unvaccinated respondents in comparison to vaccinated ones (81.6% vs. 56.5%). Significantly more HCWs, when compared to patients (64.5% vs. 58.5%), shared the opinion that the health institutions of the RS, Bosnia and Herzegovina, responded well during the pandemic, and vaccinated respondents were more likely to believe that vaccinated people experience milder clinical manifestations and have a lower risk of death than unvaccinated ones (67.3% vs. 34%). Patients (11.3% vs. 6%) and unvaccinated respondents (14.5% vs. 4.8%) more often believed that COVID-19 vaccines lead to sterility, and unvaccinated respondents (18% vs. 9.5%) more often believed that COVID-19 vaccines change the genetic material. The opinion that it is better to get sick with COVID-19 and acquire natural immunity than to receive a vaccine was shared more often by patients (61.7% vs. 49.6%) and unvaccinated respondents (74.6% vs. 42.2%). More than one-third of respondents (33.8%) were afraid of getting COVID-19, with significantly more patients than HCWs (38.3% vs. 27.4%). Patients (35.6% vs. 24.8%) and unvaccinated respondents (48.4% vs. 17.1%) were more often afraid of the vaccine than becoming infected with COVID-19 (Table 4).

### 3.5. Practice and Hesitancy Towards COVID-19 Vaccination

From all respondents vaccinated against COVID-19 (n = 315, 55.2%), HCWs were more often vaccinated in comparison to patients (70.9% vs. 44.2%). The Russian vaccine was received by 45.1% of respondents, Chinese vaccine by 29.5%, Pfizer vaccine by 21.9%, AstraZeneka vaccine by 1%, and Moderna vaccine by 2.5% of respondents. HCWs were more often vaccinated by the Russian vaccine (50.6% vs. 38.9%). Only one dose of vaccine was received by 3.8% of respondents, two doses by 57.1%, three doses by 38.4%, while four doses were received by 0.6% of respondents. The decision to receive the COVID-19 vaccine was mostly influenced by doctors’ recommendation in 62.9% of respondents. HCW were more often influenced by doctors’ recommendation to receive the vaccine when compared to patients (77.5% vs. 45.9%), while the patients’ decision was more often influenced by family members (25.3% vs. 4.1%). Fifty-four percent of vaccinated respondents stated that the main reason for their vaccination against COVID-19 was the positive effect of the vaccine on their health. HCWs, more often than patients, stated that the main reason for vaccination was demand from workplace (19.5% vs. 9.6%), while the reason such as fear of getting COVID-19 was more frequent in the patient population in comparison to HCWs (17.1% vs. 8.9%). Out of the total number of unvaccinated respondents, the most common reasons for remaining undecided and not receiving the vaccine were fear of adverse reactions to vaccines (26.8%) and the belief the vaccines were not tested enough (32). Patients, compared to HCWs, were more often afraid of adverse reaction to vaccines (35% vs. 15%), believed that the vaccines were not tested enough (40.1% vs. 20.5%), thought that they were made only for commercial profiteering (18.4% vs. 8.5%), stated distrust in pharmaceutical companies (14.5% vs. 9%), lacked confidence in their healthcare system (11.9% vs. 4.7) and reported not receiving enough information about vaccines from their doctor (12.2% vs. 8.9) (Table 5).

Table 6 shows association of socio-demographic characteristics, knowledge, and attitudes of patients and HCWs towards COVID-19 vaccination, with vaccination status as a dependent variable. The regression analyses showed that higher age (OR = 1.060, 95% CI = 1.028–1.092, *p* < 0.001), higher educational level (OR = 1.439, 95% CI = 1.019–2.033, *p =* 0.039), better knowledge that Chinese vaccines are produced in the classic way as inactivated vaccines (OR = 0.322, 95% CI = 0.149-.694, *p* = 0.044), and better knowledge that vaccinated people have milder clinical manifestations and have a lower risk of death than unvaccinated (OR = 2.329, 95% CI = 0.872–6.220, *p* = 0.004) were significantly associated with higher vaccination rate. However, the belief that the story about COVID-19 was overblown (OR = 4.052, 95% CI = 1.678–9.784, *p* = 0.002) was significantly associated with the lower COVID-19 vaccination rates (Table 6).

## 4. Discussion

Our results indicate that HCWs demonstrate a higher rate of COVID-19 vaccines acceptance, possess better knowledge about vaccines, including the technology of their production, and understand the importance of vaccines in preventing severe forms of the disease and mortality. Furthermore, HCWs had more positive attitudes towards COVID-19 vaccines. On the other hand, patients more frequently harbored misconceptions that COVID-19 vaccines alter genetic material, cause sterility, and more often believed that it is better to contract the disease than to receive the vaccine. HCWs also demonstrate better practices in COVID-19 vaccination, and among vaccinated respondents, patients more often lack trust in pharmaceutical companies, the healthcare system, and feel that they have not received enough information from their doctors. In our study, in a multivariate model, vaccination was associated with older age, higher education level, knowledge of the technology behind Chinese vaccines, and the belief that vaccinated individuals experience milder symptoms of the disease. Conversely, the belief that the COVID-19 situation was overblown was negatively associated with vaccination.

Acceptance rates of the COVID-19 vaccine among HCWs worldwide have been reported to range from 20% to 94%. Coverage in European countries (Germany, France, Poland, and Italy), Canada, Turkey, and China was high (around 79%), while Middle Eastern countries showed lower rates (below 50%) [25]. In a cross-sectional study conducted in 2021 in five hospitals in Finland among HCWs, it was found that 97% of respondents had received at least one dose of the COVID-19 vaccine, and 68% of respondents agreed that all HCWs who work in close contact with patients should be vaccinated against COVID-19 [26]. Among HCWs, there are differences in the acceptance of the COVID-19 vaccine. Dudley et al. [27] found that 94% of American HCWs reported having received at least one dose or intending to receive it soon. Vaccination was most common among pediatricians (98%), followed by family medicine doctors (96%), pharmacists (94%), and nurses (88%). When it comes to COVID-19 vaccine coverage among patients, several different studies have investigated the coverage among various patient groups. It is expected that patients would have higher vaccination coverage due to the diseases they suffer from and the importance of the vaccine in preventing mortality. This fact was highlighted by Ikeokwu et al. [28], who conducted a meta-analysis including seven studies with 21,618,297 COVID-19 patients. They found that unvaccinated patients had a 2.46 times higher chance of mortality compared to vaccinated patients. The COVID-19 vaccine acceptance rate in various studies among diabetics, patients with congenital immune disorders, patients with obstructive lung diseases, hypertension, and malignant diseases ranged from 70.9% to 88.62% for the first dose, and from 45% to 86.7% for the second dose. The highest acceptance rate was among patients with obstructive lung diseases [27,29,30,31,32,33,34]. In our study, which simultaneously determined the vaccination rate among HCWs and patients who were hospitalized at that time, the vaccination rate of HCWs with at least one dose was 70.9%, and for patients, it was 44.2% (*p* < 0.001). Among HCWs, 58% had received two doses, 39.6% received three doses, 1.8% received one dose, and only 0.6% received four doses. Among patients, 56.2% had received two doses, 37% received three doses, 6.2% received one dose, and only 0.7% received four doses. The COVID-19 vaccination coverage among our HCWs corresponds to the coverage in European countries with higher rates [25]. However, the COVID-19 vaccination coverage among our patients was lower compared to previously cited studies [27,29,30,31,32,33,34]. The differences are most likely due to the fact that our study surveyed all patients who were hospitalized at the time of the study, while other studies focused on at-risk groups of patients who were at greater risk of mortality from COVID-19. Furthermore, almost 68% of our respondents believed that the story about COVID-19 was overblown, and this belief was more often present in patients than in HCWs, and in unvaccinated compared to vaccinated individuals. Latter analyses have shown that individuals with this belief were vaccinated against COVID-19 four times less often. The present study was performed in 2024, when COVID-19 had seasonal character. The first case of COVID-19 in the Republic of Srpska, Bosnia and Herzegovina, was registered on 5 March 2020, and public health prevention measures were introduced a month earlier. Furthermore, the first major wave of COVID-19 in Bosnia and Herzegovina occurred in November 2020. This time difference between introduction of restrictive measures and appearance of COVID-19 probably contributed to the belief that the threat from COVID-19 was overblown. This may have led to a higher level of mistrust among the general population that was under restrictive measures [35].

HCWs and patients differed in their experience and presence of symptoms in the period before COVID-19 vaccination, with HCWs more often having a confirmed case of COVID-19 and presence of symptoms in comparison to patients. The higher rate of confirmed cases and symptoms in HCWa compared to patients may be explained by the fact that HCWs were more frequently exposed to SARS-CoV-2 during their worktime, while patients, in the period before COVID-19 vaccination, were under restrictive measures. Vaccinated respondents also had a confirmed case of COVID-19 more frequently, compared to unvaccinated respondents (54.9% compared to 36.7%) (*p* < 0.001). The more frequent confirmation of COVID-19 among the vaccinated can be explained by the fact that vaccinated individuals are more health-conscious, visit doctors more often and therefore get tested for COVID-19 more frequently. Additionally, COVID-19 testing was more accessible to HCWs and was more frequently conducted in hospitals, due to epidemiological measures. Differences between HCWs and patients in the COVID-19 vaccine acceptance are partly due to the knowledge about COVID-19 vaccines. Generally, HCWs and vaccinated respondents had a better level of knowledge about COVID-19 vaccines. The vaccinated respondents had better knowledge of the production technology of Chinese vaccines, and both HCWs and vaccinated respondents had better knowledge of the production technology of the Pfizer vaccine. Both HCWs and vaccinated respondents were more likely to know that vaccines protect against severe forms of the disease and mortality. Although both patients and unvaccinated respondents statistically significantly more often believed that the COVID-19 situation was blown out of proportion, it is concerning that even 61.5% of HCWs held this view. Vaccinated respondents significantly more often knew that after against COVID-19 vaccination, patients can only get mild symptoms of the disease (*p* < 0.001).

When it comes to misconceptions about vaccines, they were present among all respondents but were significantly more prevalent among unvaccinated respondents and patients. Unvaccinated individuals in our study more often believed that people died from other diseases during the pandemic, and not from COVID-19 (*p* = 0.002). Patients and unvaccinated respondents were also more often under the misconception that COVID-19 vaccines could cause sterility, despite multiple studies disproving these doubts [36,37]. Ata and colleagues included 148 studies in their research and found no evidence that COVID-19 vaccines can cause sterility. Pregnancy rates after vaccination were similar to those in unvaccinated patients [38]. Unvaccinated respondents in our study were more often under the misconception that vaccines alter human genetic material (*p* = 0.003). Patients and unvaccinated respondents more often believed that it is better to contract COVID-19 than to get vaccinated. Patients more frequently feared contracting COVID-19 (*p* = 0.007). Patients, as well as unvaccinated respondents, more often expressed fear of the vaccine rather than the disease itself. Regarding knowledge about vaccines and misconceptions about COVID-19 vaccines, similar results have been found in several studies conducted among healthcare workers, patients, and the general population samples. A study on knowledge about the COVID-19 vaccine in Spain was conducted among 316 HCWs and 389 patients. About 90% of patients and 80% of HCWs would accept vaccination in all scenarios (*p* < 0.001). Only 3–12% hesitated about the COVID-19 vaccine. Compared to 40–70% of patients, 60–80% of HCWs perceived a high risk of COVID-19 (*p* < 0.001). HCWs had better knowledge and perception of the risks of COVID-19 compared to the surveyed patients, but they had a higher percentage of hesitation to receive the COVID-19 vaccine [18], which is contrary to our study. Although a cross-sectional study conducted among 400 Pakistani respondents showed poor knowledge (50.5%) and negative perception towards COVID-19 vaccination, the attitude towards vaccination was correct (75.1%) [39]. Research conducted by Venkataraman et al. [40] in the general rural population of India showed that 81.71% of respondents had adequate knowledge, and 81.5% had a positive attitude towards the COVID-19 vaccine. Women (85.3%) had a more positive attitude than men (77%). Respondents with a positive attitude (86.9%) had a higher level of knowledge about the COVID-19 vaccine than respondents with a negative attitude (57.8%). In our study, there were no differences in knowledge and attitudes towards COVID-19 vaccination by gender. The findings of the study in Bangladesh reflect inadequate knowledge but more positive attitudes towards the COVID-19 vaccine among the general population in Bangladesh [41]. According to research conducted by Dudley and colleagues [27], nearly half of unvaccinated HCWs (47%) were concerned about side effects, and one-third of unvaccinated HCWs (33%) were concerned that the vaccine was developed too quickly. Among our respondents, the decision to get vaccinated was most influenced by doctors’ advice, particularlyamong HCWs (77.5%). Another important influence factor were family members, especially among patients. Only 1.2% of HCWs and 2.7% of patients stated that they made the decision to get vaccinated based on information from social media. In a study conducted in Italy, the majority of respondents (92.6%) had received at least two doses of the SARS-CoV-2 vaccine. Most respondents (79.2%) stated that the decision to get vaccinated was their own choice, while only 4.3% were convinced by a general practitioner or occupational medicine doctor [42].

When it comes to hesitancy about vaccination in our study, it was more common among patients and related to fear of adverse reactions to the vaccine, concern that the vaccine was not sufficiently tested, the belief that vaccines were made only for profit, lack of trust in pharmaceutical companies, lack of trust in the healthcare system, or not having received enough information from their doctor. Among HCWs, the most common concerns were the belief that vaccines were not sufficiently tested and fear of adverse reactions to the vaccines. In a cross-sectional study conducted among 10,471 adults in Bosnia and Hezegovina, 74.3% of respondents were hesitant or completely rejected COVID-19 vaccination. Authors stated that the main reason of hesitancy and refusal of vaccination was deficiency of clinical data (30.2%) [43]. In a neighboring country Serbia, Markovic-Denic et al. [44] demonstrated in their panel study that in 2020, 64.4% of respondents believed a future COVID-19 vaccine could provide protection, 9.7% thought it could not, and 25.9% were uncertain. One year later, positive attitudes slightly dropped from 64.7% to 62.5%. However, 34% of those with negative views became supportive, while 28.9% shifted to uncertainty about the vaccine’s effectiveness (*p* < 0.001). These studies [43,44] indicate somewhat more negative attitudes compared to our study, which can be explained by the fact that this study [43] was conducted before COVID-19 vaccines were introduced. Additionally, in a study in Finland, among 5120 doctors and nurses working in five hospitals with close patient contact, hesitancy was low but most often due to fear of side effects from the vaccines [26]. In the study conducted in Italy, the main factor for hesitancy was using Facebook as the primary source of information, while predictors of acceptance included younger age, close contact with high-risk groups, and receiving the flu vaccine during the 2019/2020 season. Reasons for hesitation also included a lack of trust in the safety of the vaccine (85%) and receiving little (78%) or contradictory (69%) information about vaccines [45]. According to research among patients with diabetes mellitus in Sub-Saharan Africa, the main reasons for not receiving COVID-19 vaccines were identified as: advice from religious leaders, concerns about the safety, effects, and efficacy of vaccines, distrust in pharmaceutical companies, conspiracy theories about vaccines, and personal beliefs. However, participants would get vaccinated if they had a higher level of knowledge about it, received positive feedback from vaccinated individuals, were rewarded for getting the vaccine, and if vaccination was a prerequisite for travel [12]. In our study, in a multivariate model, vaccination was associated with older age, higher education level, knowledge of the technology behind Chinese vaccines, and the belief that vaccinated individuals experience milder symptoms of the disease. Conversely, the belief that the COVID-19 situation was overblown was negatively associated with vaccination, with those holding this belief being about four times less likely to get vaccinated (OR = 4.052; 95% CI = 1.678–9.784; *p* = 0.002). Respondents who believed that vaccinated individuals can only get mild symptoms were 2.3 times more likely to get vaccinated against COVID-19. Results from other studies indicate similar findings regarding factors leading to vaccination. In a study conducted by Lucane et al. [31], age was associated with vaccination status, with younger patients being significantly less likely to be vaccinated (U = 8585, *p* < 0.001). In a study conducted among 1135 Greek, Spanish, Cypriot, Albanian, and Kosovar nurses, it was shown that the key factors for willingness to get vaccinated were male gender, living in a country with a high mortality rate, high level of knowledge about vaccines, and having received the flu vaccine in the last two years [46]. Similar predictors of willingness to get vaccinated against COVID-19 were found in a study conducted by Cordina et al. [47]: the belief that the vaccine protects health, the respect for healthcare workers’ advice, and having received the flu vaccine in the previous year. In our study, among those who regularly receive the flu vaccine, there were differences in favor of patients, but we could not test the variable in the multivariate logistic regression model because we only collected this variable for vaccinated individuals, which represents a limitation of the study. According to a study in Bangladesh, good vaccination practices were associated with older age (AOR: 1.52, 95% CI: 1.10 to 2.11, *p* = 0.01), higher education (AOR: 2.78, 95% CI: 1.58 to 4.89, *p* < 0.001), and anxiety [48]. According to research in Jordan among medical and non-medical respondents, the biggest factor leading to vaccine refusal was rumors on social media about the vaccine’s safety. These rumors were not believed by 56.8% of HCWs and 35.1% of non-medical respondents. Advice from medical staff (OR = 0.83; 95% CI = 0.70 to 0.98; *p* = 0.026) and social media (OR = 1.21; 95% CI = 1.04 to 1.41; *p* = 0.012) were significantly associated with the willingness to accept the vaccine [21]. Most studies around the world have reported similar findings regarding knowledge, attitudes, and practices regarding COVID-19 vaccination. Some studies were conducted among patients, some among HCWs, and most of them were conducted over different time periods. Vaccination knowledge, attitudes and practices can change over time. The strength of our study is that we conducted the research simultaneously, among both patients and HCWs. The main findings indicate that the majority of our respondents trust health professionals when making decisions about vaccination. At the same time, our study reveals that there are also misconceptions among HCWs about COVID-19 vaccines, which indirectly points to the right path in increasing the COVID-19 vaccination coverage. It certainly refers to the education of HCWs. The epidemiological situation of COVID-19 currently indicates a sporadic occurrence in Bosnia and Herzegovina, but we are threatened by similar pandemics in the future, which requires better preparedness and a better response.

At the end of 2020, a national study was conducted by our authors about seroprevalence of SARS-CoV-2 antibodies and knowledge, attitudes and practice toward COVID-19 in the Republic of Srpska, Bosnia and Herzegovina [49], and the results showed that the seroprevalence rate of SARS-CoV-2 antibodies was 40.3%, and about 50% of respondents from the general population stated that the COVID-19 infection can be prevented by receiving the COVID-19 vaccine. During the conduct of this study and in their practical work on suppressing the COVID-19 pandemic, some authors encountered frequent negative attitudes towards COVID-19 vaccination among HCWs. In the context of drawing conclusions for the resolution of future similar pandemics, the authors wanted to check whether there had been a change in attitudes towards COVID-19 vaccination, and this is the main reason why this study was performed in 2024. In the research, significant data useful for public health practice was obtained, but there are also certain limitations in the study. The same questionnaire was used for both patients and HCWs, which prevented the inclusion of some specific questions that would differ for these two groups of respondents.

## 5. Conclusions

HCWs had better knowledge, more positive attitudes, and better COVID-19 vaccination practices. However, there are still certain dilemmas and hesitations among HCWs. Independent predictors of COVID-19 vaccination were older age, higher education level, knowledge of vaccine production technology, and the belief that vaccinated individuals have milder symptoms of the disease, while the belief that the COVID-19 situation was overblown was negatively associated with vaccination. In preparation for responding to future similar epidemics, it is necessary to conduct training for HCWs, who must find different channels of interpersonal communication to improve vaccine acceptance. It is necessary to apply the obtained results in a form of HCWs’ education, and in the context of the discussion about the identified misconceptions about COVID-19 vaccination.

## Figures and Tables

**Table 1:** Process of selecting the study sample.

Hospital	Number of Patients on the Day of the Study	Number of Patients Meeting the Inclusion Criteria	Number of Patients Who Agreed to Participate in the Study	Number of HCWs Working on the Day of the Study	Number of HCWs Meeting the Inclusion Criteria	Number of HCWs Who Agreed to Participate in the Study
Doboj	210	185	129	201	99	69
Zvornik	119	81	67	134	70	41
Istočno Sarajevo	109	69	55	130	63	43
Foča	85	63	41	150	75	43
Trebinje	70	55	45	120	60	38
Total	593	453	337	735	367	234

HCWs—healthcare workers.

**Table 2:** The differences in socio-demographic characteristics between respondents according to the study sample and COVID-19 vaccination status.

Variables	Study Sample n (%)		*p*	Vaccination Status n (%)		*p*	Total (n = 571, 100%) n (%)
	Patients (n = 337, 59%)	HCW (n = 234, 41%)		No (n = 256, 44.8%)	Yes (n = 315, 55.2%)		
**Gender**							
Male	129 (38.3)	52 (22.2)	**<0.001** *	85 (33.2)	96 (30.5)	0.486 *	181 (31.7)
Female	208 (61.7)	182 (77.8)		171 (66.8)	219 (69.5)		390 (68.3)
**Age** (M ± SD)	37.15 ± 15.91	42.10 ± 12.29	**<0.001** **	33.59 ± 13.30	43.72 ± 14.28	**<0.001** **	39.18 ± 14.73
**Age groups**							
19 to 45 years	238 (70.6)	146 (62.4)	**0.001** *	205 (80.1)	179 (56.8)	**<0.001** *	384 (67.3)
45 to 64 years	79 (23.4)	84 (35.9)		48 (18.8)	115 (36.5)		163 (28.5)
65 to 81 years	20 (5.9)	4 (1.7)		3 (1.2)	21 (6.7)		24 (4.2)
**Education**							
Primary school	9 (2.7)	2 (0.9)	**0.002** *	4 (1.6)	7 (2.2)	**0.015** *	11 (1.9)
High school	201 (59.6)	111 (47.4)		157 (61.3)	155 (49.2)		312 (54.6)
University degree	114 (33.8)	116 (49.6)		91 (35.5)	139 (44.1)		230 (40.3)
Master	9 (2.7)	2 (0.9)		1 (0.4)	10 (3.2)		11 (1.9)
PhD	4 (1.2)	3 (1.3)		3 (1.2)	4 (1.3)		7 (1.2)
**Financial monthly income**							
Low	82 (24.3)	55 (23.5)	0.584 *	66 (25.8)	71 (22.5)	0.597 *	137 (24.0)
Lower-middle	162 (48.1)	119 (50.9)		120 (46.9)	161 (51.1)		281 (49.2)
Upper-middle	79 (23.4)	55 (23.5)		63 (24.6)	71 (22.5)		134 (23.5)
High	14 (4.2)	5 (2.1)		7 (2.7)	12 (3.8)		19 (3.3)
**Place of living**							
Rural	62 (18.4)	43 (18.4)	0.996 *	55 (21.5)	50 (15.9)	0.085 *	105 (18.4)
Urban	275 (81.6)	191 (81.6)		201 (78.5)	265 (84.1)		466 (81.6)

HCW—healthcare workers; M—mean; ± SD—standard deviation; *p*—statistical significance was measured by χ^2^—chi square test and * Mann–Whitney test **; significant values are bolded.

**Table 3:** Experience with COVID-19 among study sample (patients and HCWs) and vaccination status.

Variables	Study Sample n (%)		*p*	Vaccination Status n (%)		*p*	Total (n = 571, 100%) n (%)
	Patients (n = 337, 59%)	HCWs (n = 234, 41%)		No (n = 256, 44.8%)	Yes (n = 315, 55.2%)		
**Confirmed cases of COVID-19**							
Not confirmed	197 (58.5)	107 (45.7)	**0.003** *	162 (63.3)	142 (45.1)	**<0.001** *	304 (53.2)
Confirmed	140 (41.5)	127 (54.3)		94 (36.7)	173 (54.9)		267 (46.8)
**Type of test used for confirmation of COVID-19 cases (n = 267)**							
Antigen test	53 (37.9)	36 (28.3)	0.118 *	32 (34.0)	57 (32.9)	**0.012** *	89 (33.3)
Serological testing	12 (8.6)	19 (15.0)		18 (19.1)	13 (7.5)		31 (11.6)
PCR test	75 (53.6)	72 (56.7)		44 (46.8)	103 (59.5)		147 (55.1)
**Presence of symptoms before COVID-19 vaccination (n = 503)**							
No	175 (55.6)	76 (40.4)	**0.001** *	135 (59.0)	116 (42.3)	**<0.001** *	251 (49.9)
Yes	140 (44.4)	112 (59.6)		94 (41.0)	158 (57.7)		252 (50.1)
**Confirmed COVID-19 happened (n = 267)**							
Before vaccination	102 (72.9)	87 (68.5)	0.435 *	68 (72.3)	121 (69.9)	0.681 *	189 (70.8)
After vaccination	38 (27.1)	40 (31.5)		26 (27.7)	52 (30.1)		78 (29.2)
**Severity of COVID-19 clinical manifestations (n = 267)**							
Mild	83 (59.3)	79 (62.2)	0.316 *	62 (66.7)	100 (57.5)	0.265 *	162 (60.7)
Moderate	50 (35.7)	37 (29.1)		27 (29.0)	60 (34.5)		87 (32.6)
Severe	7 (5.0)	11 (8.7)		4 (4.3)	14 (8.0)		18 (6.7)
**Hospitalization because of COVID-19 (n = 267)**							
No	121 (85.8)	112 (88.9)	0.452 *	82 (88.2)	151 (86.9)	0.758 *	233 (87.3)
Yes	20 (14.2)	14 (11.1)		11 (11.8)	23 (13.1)		34 (12.7)
**Hospital days (n = 34) (M ± SD)**	2.69 ± 3.21	2.39 ± 3.15	0.231 **	1.82 ± 2.11	1.76 ± 2.73	0.274 **	2.54 ± 3.18
**Family member had COVID-19 infection**							
No	134 (39.8)	84 (35.9)	0.350 *	105 (41.0)	113 (35.9)	0.208 *	218 (38.2)
Yes	203 (60.2)	150 (64.1)		151 (59.0)	202 (64.1)		353 (61.8)
**Hospitalization of family members**							
No	287 (85.2)	193 (82.5)	0.389 *	214 (83.6)	266 (84.4)	0.782 *	480 (84.1)
Yes	50 (14.8)	41 (17.5)		42 (16.4)	49 (15.6)		91 (15.9)
**Hospitalization of colleagues/friends**							
No	45 (13.4)	24 (10.3)	0.264 *	33 (12.9)	36 (11.4)	0.594 *	69 (12.1)
Yes	292 (86.6)	210 (89.7)		223 (87.1)	279 (88.6)		502 (87.9)
**If you know someone who died from COVID-19, were they?**							
Family members (Yes)	34 (10.1)	25 (10.7)	0.818 *	23 (9.0)	36 (11.4)	0.340 *	59 (10.3)
Colleagues (Yes)	19 (5.6)	28 (12.0)	**0.007** *	14 (5.5)	33 (10.5)	**0.030** *	4 (8.2)
Acquaintances (Yes)	229 (68.0)	184 (78.6)	**0.005** *	172 (67.2)	241 (76.5)	**0.013** *	413 (72.3)

HCWs—healthcare workers; M—mean; ± SD—standard deviation; *p*—statistical significance was measured by χ^2^—chi square test and * Mann–Whitney test **, significant values are bolded.

**Table 4:** Knowledge and attitudes about vaccination against COVID-19 among study sample (patients and HCWs) and vaccination status.

Variables	Study Sample n (%)		*p*	Vaccination Status n (%)		*p*	Total (n = 571, 100%) n (%)
	Patients (n = 337, 59%)	HCWs (n = 234, 41%)		No (n = 256, 44.8%)	Yes (n = 315, 55.2%)		
**Knowledge about COVID-19 vaccination (yes)**							
Knowledge of availability of various vaccines against COVID-19 in the RS							
Russian vaccine (Sputnik V)	316 (93.8)	216 (92.3)	0.496	238 (93.0)	294 (93.3)	0.864	532 (93.2)
Chinese vaccine (Sinopharm, Sinovac)	297 (88.1)	211 (90.2)	0.444	225 (87.9)	283 (89.8)	0.459	508 (89.0)
Pfizer vaccine (BNT162b2)	302 (89.6)	202 (86.3)	0.230	230 (89.8)	274 (87.0)	0.291	504 (88.3)
Moderna vaccine (mRNA-1273)	199 (59.1)	127 (54.3)	0.257	143 (55.9)	183 (58.1)	0.591	326 (57.1)
AstraZeneca vaccine (AZD122)	236 (70.0)	152 (65.0)	0.201	175 (68.4)	213 (67.6)	0.850	388 (68.0)
All before mentioned vaccines	194 (57.6)	124 (53.0)	0.279	139 (54.3)	179 (56.8)	0.545	318 (55.7)
Chinese vaccines are produced in the classic way as inactivated vaccines	198 (58.8)	142 (60.7)	0.888	133 (52.0)	207 (65.7)	**<0.001**	340 (59.5)
Pfizer vaccine uses RNA technology to induce immune response	122 (36.2)	120 (51.3)	**<0.001**	94 (36.7)	148 (47.0)	**0.006**	242 (42.4)
Pfizer and Moderna vaccines lead to the best immune response against COVID-19	75 (22.3)	64 (7.4)	0.391	49 (19.1)	90 (28.6)	**<0.001**	139 (24.3)
Vaccines against COVID-19 protect against severe forms of disease and death	231 (68.5)	189 (80.8)	**0.001**	173 (67.6)	247 (78.4)	**0.006**	420 (73.6)
Around 7.000 people died from COVID-19 until December 2023. in RS	112 (33.2)	78 (33.3)	0.982	92 (35.9)	98 (31.1)	0.157	190 (33.3)
**Attitudes about COVID-19 vaccination (yes)**							
The story about COVID-19 is overblown	243 (72.1)	144 (61.5)	**0.009**	209 (81.6)	178 (56.5)	**<0.001**	387 (67.8)
During pandemic people were dying from other diseases, not from COVID-19	98 (29.1)	49 (20.9)	0.091	82 (32.0)	65 (20.6)	**0.002**	147 (25.7)
The health institutions of the RS responded well during the pandemic	197 (58.5)	151 (64.5)	**<0.001**	159 (62.1)	189 (60.0)	0.943	348 (60.9)
Vaccinated people have milder clinical manifestations and die less often than unvaccinated	164 (48.7)	135 (57.7)	0.158	87 (34.0)	212 (67.3)	**<0.001**	299 (52.4)
COVID-19 vaccines lead to the sterility in young	38 (11.3)	14 (6.0)	**0.031**	37 (14.5)	15 (4.8)	**<0.001**	52 (9.1)
COVID-19 vaccines change the genetic material of the vaccinated individuals	49 (14.5)	27 (11.5)	0.299	46 (18.0)	30 (9.5)	**0.003**	76 (13.3)
Severe adverse reactions to COVID-19 vaccines are very rare	82 (24.3)	62 (26.5)	0.558	57 (22.3)	87 (27.6)	0.143	144 (25.2)
It is better to get sick from COVID-19 and acquire natural immunity than to receive a vaccine	208 (61.7)	116 (49.6)	**0.004**	191 (74.6)	133 (42.2)	**<0.001**	324 (56.7)
I was afraid of getting COVID-19	129 (38.3)	64 (27.4)	**0.007**	76 (29.7)	117 (37.1)	0.061	193 (33.8)
I am more afraid of the vaccine than getting the COVID-19	120 (35.6)	58 (24.8)	**0.006**	124 (48.4)	54 (17.1)	**<0.001**	178 (31.2)

HCWs—healthcare workers; RS—Republic of Srpska, *p*—statistical significance was measured by χ^2^—chi square test; significant values are bolded.

**Table 5:** Practice and hesitancy towards vaccination against COVID-19 among study sample (patients and HCWs).

Variables	Study Sample n (%)		*p*	Total n (%)
	Patients (n = 337, 59%)	HCW (n = 234, 41%)		
**Practice towards COVID-19 vaccination**				
**I am vaccinated against COVID-19 (n = 315)**	149 (44.2)	166 (70.9)	**<0.001**	315 (55.2)
Russian vaccine (Sputnik V)	58 (38.9)	84 (50.6)	**<0.001**	142 (45.1)
Chinese vaccine (Sinopharm, sinovac)	42 (28.2)	51 (30.7)	0.751	93 (29.5)
Pfizer vaccine (BNT162b2)	38 (25.5)	31 (18.7)	0.482	69 (21.9)
Moderna vaccine (mRNA-1273)	3 (2.0)	0 (0.0)	0.711	3 (1.0)
AstraZeneca vaccine (AZD122)	8 (5.4)	0 (0.0)	0.823	8 (2.5)
**Number of doses of vaccines received (n = 315)**				
One dose	9 (6.2)	3 (1.8)	0.245	12 (3.8)
Two doses	82 (56.2)	98 (58.0)	0.375	180 (57.1)
Three doses	54 (37.0)	67 (39.6)	0.472	121 (38.4)
Four doses	1 (0.7)	1 (0.6)	0.969	2 (0.6)
**The decision to receive the vaccine was mostly influenced by (n = 315)**				
Doctors’ recommendation	67 (45.9)	131 (77.5)	**<0.001**	198 (62.9)
Friends’ recommendation	13 (8.9)	10 (5.9)	0.659	23 (7.3)
Influence by family members	37 (25.3)	7 (4.1)	**<0.001**	44 (14.0)
Media (TV and internet)	25 (17.1)	19 (11.2)	0.108	44 (14.0)
Social networks	4 (2.7)	2 (1.2)	0.621	6 (1.9)
**Reason for COVID-19 vaccination (n = 315)**				
It has a positive effect on my health	75 (51.4)	95 (56.2)	0.019	170 (54.0)
The country I traveled to demanded it	17 (11.6)	12 (7.1)	0.103	29 (9.2)
It was demanded by my workplace	14 (9.6)	33 (19.5)	**<0.001**	47 (14.9)
I feared to get COVID-19	25 (17.1)	15 (8.9)	**0.022**	40 (12.7)
Others in my environment also got vaccinated	15 (10-3)	14 (8.3)	0.278	29 (9.2)
**I regularly receive the flu vaccine (n =571)**	93 (27.6)	30 (12.8)	**<0.001**	123 (21.5)
**Hesitancy towards COVID-19 vaccination (n = 256)**				
**The reasons I remained undecided and did not receive the COVID-19 vaccine is:**				
Fear from adverse reactions to vaccines	118 (35.0)	35 (15.0)	**<0.001**	153 (26.8)
They were not tested enough	135 (40.1)	48 (20.5)	**<0.001**	183 (32.0)
They are made only for profiteering	62 (18.4)	20 (8.5)	**0.001**	82 (14.4)
I don’t trust pharmaceutical companies	49 (14.5)	21 (9.0)	**0.046**	70 (12.3)
I do not believe in our Ministry of Health	30 (8.9)	11 (4.7)	0.056	41 (7.2)
I don’t have confidence in our healthcare system	40 (11.9)	11 (4.7)	**0.003**	51 (8.9)
I did not get enough information from my doctor	41 (12.2)	15 (8.9)	**0.023**	56 (9.8)

HCWs—healthcare workers; *p*—statistical significance was measured by χ^2^—chi square test; significant values are bolded.

**Table 6:** Association of socio-demographic characteristics, knowledge, attitudes, and hesitancy of patients and HCWs towards COVID-19 vaccination rate.

Variables	B	OR	95% CI	*p*
Age	0.058	1.060	1.028–1.092	**<0.001**
Education level	0.364	1.439	1.019–2.033	**0.039**
PCR testing	−1.628	0.196	0.033–1.158	0.072
Confirmed cases of COVID-19	−0.016	0.984	0.662–1.464	0.938
Presence of symptoms before vaccination against COVID-19	0.064	1.066	0.469–2.423	0.879
Colleagues died from COVID-19	−0.098	0.906	0.301–2.728	0.861
Acquaintances died from COVID-19	−0.049	0.952	0.410–2.211	0.909
Chinese vaccines are produced in the classic way as inactivated vaccines	1.134	0.322	0.149–0.694	**0.004**
Pfizer vaccine uses RNA technology to induce immune response	0.146	1.158	0.547–2.448	0.702
Pfizer and Moderna vaccines lead to the best immune response against COVID-19	0.845	0.517	0.171–1.366	0.092
Vaccines against COVID-19 protect against severe forms of disease and death	−0.747	0.474	0.203–1.109	0.085
The story about COVID-19 is overblown	−1.399	4.052	1.678–9.784	**0.002**
During pandemic people were dying from other diseases, not from COVID-19	0.096	1.101	0.470–2.578	0.824
Vaccinated people have a milder clinical manifestations and die much less often than unvaccinated	1.692	2.329	0.872–6.220	**0.004**
COVID-19 vaccines lead to the sterility in young	−1.085	0.306	0.083–1.231	0.074
COVID-19 vaccines change the genetic material of the vaccinated individuals	0.700	2.013	0.738–5.490	0.172
It is better to get sick from COVID-19 and acquire natural immunity than to receive a vaccine	0.322	1.379	0.575–3.306	0.471
I am more afraid of the vaccine than getting COVID-19	0.070	1.073	0.475–2.425	0.866
Constant	−0.832	0.435	/	0.662

HCWs—healthcare workers; RS—Republic of Srpska; B—unstandardized regression coefficient; OR—odds ratio; 95% CI—confidence interval; *p*—statistical significance; significant values are bolded.

## Data Availability

The original contributions presented in this study are included in the article. Further inquiries can be directed to the corresponding author.
